# Comprehensive Exploration of Antinuclear Antibodies (ANAs): Unveiling Clinical Significance, Associations with Cancer, and the Nuances of Differential Diagnosis in Positive ANA Patients

**DOI:** 10.3390/diagnostics14030320

**Published:** 2024-02-01

**Authors:** Krasimir Kraev, Bozhidar Hristov, Petar Uchikov, Maria Kraeva, Yordanka Basheva-Kraeva, Siyana Valova, Maria Koleva-Ivanova, Stanislava Popova-Belova, Milena Sandeva, Dzhevdet Chakarov, Mariela Geneva-Popova

**Affiliations:** 1 Department of Propaedeutics of Internal Diseases “Prof. Dr. Anton Mitov”, Faculty of Medicine, Medical University of Plovdiv, 4002 Plovdiv, Bulgaria; spopova92@abv.bg (S.P.-B.); genevapopova@yahoo.com (M.G.-P.); 2 Second Department of Internal Diseases, Section “Gastroenterology”, Medical Faculty, Medical University of Plovdiv, 4002 Plovdiv, Bulgaria; hristov.bozhidar@abv.bg; 3 Department of Special Surgery, Faculty of Medicine, Medical University of Plovdiv, 4002 Plovdiv, Bulgaria; puchikov@yahoo.com; 4 Department of Otorhinolaryngology, Medical Faculty, Medical University of Plovdiv, 4002 Plovdiv, Bulgaria; kraevamaria93@gmail.com; 5 Department of Ophthalmology, Medical Faculty, Medical University of Plovdiv, 4002 Plovdiv, Bulgaria; 6 Second Department of Internal Diseases, Section “Nephrology”, Medical Faculty, Medical University of Plovdiv, 4002 Plovdiv, Bulgaria; siyanavalova@abv.bg; 7 Department of General and Clinical Pathology, Faculty of Medicine, Medical University of Plovdiv, 4000 Plovdiv, Bulgaria; mariya.kolevaivanova@gmail.com; 8 Department of Midwifery, Faculty of Public Health, Medical University of Plovdiv, 4000 Plovdiv, Bulgaria; sandewa@abv.bg; 9 Department of Propaedeutics of Surgical Diseases, Section of General Surgery, Faculty of Medicine, Medical University of Plovdiv, 4002 Plovdiv, Bulgaria

**Keywords:** antinuclear antibodies, autoimmune rheumatic diseases, cancer associations

## Abstract

This comprehensive review delves into the complex realm of antinuclear antibodies (ANAs), expanding beyond their traditional involvement in autoimmune rheumatic disorders. By digging into historical changes, diagnostic complexity, and clinical significance, the debate reveals the shifting relationships between ANAs, particularly with cancer. Specialized studies provide practical insights on ANA testing processes, standardization, and upcoming challenges. Examining prevalence trends in the United States provides a time dimension to ANA dynamics, linking autoimmune and oncological considerations. The debate delves into the complexity of lupus erythematosus, emphasizing ANAs’ diverse presentations and their potential as flexible diagnostic and prognostic indicators. The complex relationship between ANAs and cancer is highlighted, demonstrating their potential as early markers or indicators of malignancies. Looking ahead, this synthesis anticipates advances in personalized medicine and collaborative research, putting ANAs at the forefront of advanced diagnostics and treatments for autoimmune disorders and cancer. This synthesis envisions a future for ANA research in which these antibodies play a critical role in promoting personalized treatment, enhancing diagnostics, and fostering collaborative initiatives that cross traditional boundaries. As ANAs grow more prominent at the junction of autoimmune illnesses and cancer, this synthesis lays the path for further research and novel advances in understanding, diagnosing, and treating complicated medical conditions.

## 1. Introduction

**Figure 1:**
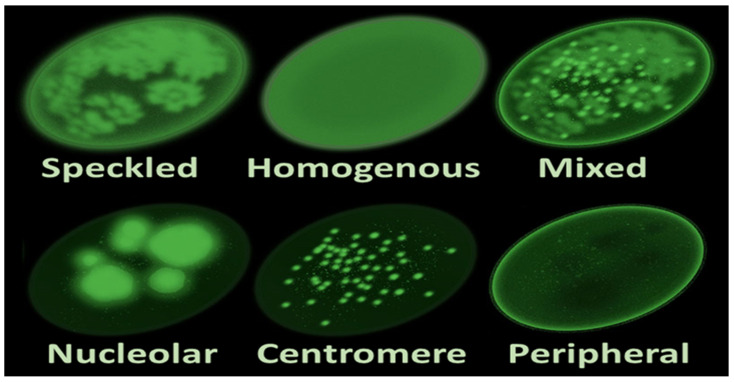
Main antinuclear antibody patterns on immunofluorescence [3].

The discovery of antinuclear antibodies (ANAs) in the mid-20th century during studies on systemic lupus erythematosus (SLE) marked a significant breakthrough [1]. The identification of a luminous staining pattern associated with ANAs in 1957 led to further investigations in the 1970s and 1980s, confirming their connection to various autoimmune disorders, expanding beyond SLE to include conditions such as rheumatoid arthritis and systemic sclerosis [2,3]. ANA testing, utilizing methodologies like ELISA and immunoblotting, has since become an indispensable diagnostic tool in the field, providing valuable insights into the diverse patterns that emerge (Figure 1) [1,2,3].

Integral to immunological investigations, ANAs offer profound insights into the intricate diagnostic landscape of autoimmune disorders, specifically targeting components within the cell nucleus. Detected through sophisticated laboratory techniques like indirect immunofluorescence (IIF) or enzyme-linked immunosorbent assay (ELISA) [1], ANAs play a crucial role in enhancing the accuracy of diagnostic procedures. Despite their association with autoimmune disorders, it is essential to recognize that not all individuals with positive ANA results definitively have these conditions. Positive ANA results, when considered alongside clinical symptoms and other tests, contribute to a comprehensive diagnostic process [2,3]. Ongoing research aims to deepen our understanding of ANAs and improve diagnostic techniques and therapeutic approaches for autoimmune disorders.

This review provides a thorough examination of the clinical aspects related to antinuclear antibodies (ANAs), highlighting their crucial significance in the diagnosis and treatment of autoimmune disorders. These antibodies are important indicators, especially in connective tissue illnesses. They are not just used for diagnosis, but also help guide treatment decisions and estimate prognosis. The complex correlation between ANAs and different health disorders, such as cancer, highlights the broadening range of their clinical significance. Furthermore, through the integration of findings from specialized studies and the identification of new difficulties, this review not only offers a comprehensive overview of current knowledge but also highlights the dynamic character of ANA research. This study is an important and useful resource for doctors, researchers, and laboratory professionals due to the growing prevalence trends and the urgent need for standardization in testing techniques.

## 2. Clinical Significance of ANAs: Decoding the Diagnostic Symphony

At the nexus of autoimmune rheumatic diseases (ARDs), antinuclear antibodies (ANAs) emerge as crucial sentinels, orchestrating a diagnostic symphony that unfolds within the realms of immunological intricacy. These antibodies, with their propensity to target nuclear and cytoplasmic components, unfurl distinctive fluorescence patterns that serve as invaluable clues in the diagnostic odyssey. While their presence is often synonymous with systemic lupus erythematosus (SLE), Sjögren’s syndrome (SS), and scleroderma, decoding ANA patterns unlocks a nuanced understanding of associated autoimmune diseases [3,4,5].

The diagnostic landscape is enriched by the variety of patterns ANAs present under the microscopic lens. Homogeneous, speckled, centromeric, and nucleolar patterns stand as the building blocks of this diagnostic language. A homogenous pattern, often indicative of antibodies against double-stranded DNA (dsDNA), is a hallmark of SLE. Speckled patterns, on the other hand, may suggest antibodies against extractable nuclear antigens (ENAs) and are frequently associated with a spectrum of autoimmune conditions [5].

Centromere patterns, portraying a distinctive speckling at the centromeres of chromosomes, are strongly linked with limited cutaneous systemic sclerosis (lcSSc) and CREST syndrome (calcinosis, Raynaud’s phenomenon, esophageal dysmotility, sclerodactyly, and telangiectasia). Nucleolar patterns, characterized by a bright staining of the nucleoli, are often associated with scleroderma and systemic sclerosis [6].

However, the diagnostic narrative extends beyond these patterns. The clinical significance of ANAs resides not only in their presence, but in the specific patterns they weave. Understanding the intricate dance of these antibodies is akin to deciphering a diagnostic code that directs clinicians toward accurate disease classification [4].

While ANAs are considered hallmark autoantibodies, their diagnostic specificity can be challenging. A positive ANA result prompts the need for further investigations to unveil the specificities within this diagnostic umbrella. Clinicians, armed with a discerning eye, delve into the patient’s clinical history, symptoms, and specialized laboratory tests to differentiate between autoimmune and non-autoimmune conditions [7].

The diagnostic prowess of ANAs extends beyond disease classification. These antibodies, acting as beacons in the diagnostic landscape, provide valuable information about disease prognosis, potential complications, and treatment responses. In conditions like SLE, where ANAs are almost omnipresent, the specific patterns and titers aid in delineating the disease severity and guiding therapeutic strategies [8]. The clinical significance of ANAs becomes even more pronounced in the realm of autoimmune liver diseases. Primary biliary cholangitis (PBC) and autoimmune hepatitis (AIH) often exhibit distinctive ANA patterns, contributing to accurate disease identification and subsequent management. The nuanced understanding of these patterns plays a pivotal role in guiding therapeutic decisions and predicting disease progression [9,10].

In conclusion, the clinical significance of ANAs extends beyond their status as diagnostic markers. They embody a diagnostic symphony, each pattern and specificity composing a unique melody that guides clinicians through the intricate landscape of autoimmune diseases. Decoding this symphony requires not only technical precision in laboratory testing but a nuanced understanding of the clinical context. As research continues to unveil the intricacies of ANAs, their role in autoimmune disease diagnostics promises to evolve, offering clinicians more refined tools for accurate disease identification and personalized therapeutic interventions.

## 3. Differential Diagnosis in Positive ANA Patients: Navigating Complexity

A positive antinuclear antibody (ANA) test result is a diagnostic starting point that requires careful consideration of various factors to elucidate the underlying health condition. The presence of ANAs in an individual’s serum indicates an immune system response targeting the body’s own cell nuclei, often associated with autoimmune diseases. However, the interpretation of a positive ANA test is not straightforward, and a comprehensive approach to the differential diagnosis is crucial for accurate clinical decision-making.

### 3.1. Connective Tissue Diseases (CTDs)

Positive ANA findings are strongly associated with a spectrum of connective tissue diseases, serving as a hallmark for conditions like systemic lupus erythematosus (SLE), systemic sclerosis (SSc), rheumatoid arthritis (RA), and Sjögren’s syndrome. The distinct ANA patterns observed through immunofluorescence on HEp-2 cells aid in narrowing down the specific CTD. For instance, a speckled pattern is commonly linked to SLE, while a centromere pattern is indicative of limited cutaneous SSc [11,12,13].

### 3.2. Systemic Lupus Erythematosus (SLE)

SLE, a prototypical autoimmune disease, often manifests with a positive ANA result. However, a positive ANA result alone does not confirm SLE, as these antibodies can also be present in other autoimmune conditions. Diagnostic criteria, including clinical manifestations and additional specific autoantibodies such as anti-double-stranded DNA (anti-dsDNA) and anti-Smith (anti-Sm) antibodies, are crucial for a definitive SLE diagnosis [14,15].

### 3.3. Rheumatoid Arthritis (RA)

While RA is primarily characterized by the presence of rheumatoid factor and anti-citrullinated protein antibodies (ACPA), some RA patients may exhibit positive ANA results. The coexistence of ANAs in RA can contribute to joint inflammation and complicate the overall clinical picture, necessitating a comprehensive assessment for optimal management [16].

### 3.4. Sjögren’s Syndrome

Positive ANA findings, particularly with a speckled pattern, are common in Sjögren’s syndrome. However, the distinctive involvement of exocrine glands, leading to symptoms such as dry eyes and dry mouth, is pivotal for confirming the diagnosis. Additional testing for anti-Ro (SSA) and anti-La (SSB) antibodies further supports the identification of Sjögren’s syndrome [17].

### 3.5. Systemic Sclerosis (SSc)

Systemic sclerosis, or scleroderma, often presents with a positive ANA result, and specific ANA patterns like a nucleolar or centromere pattern can guide towards the limited or diffuse form of the disease. However, clinical manifestations, including skin thickening and internal organ involvement, are vital for accurate classification and management [18,19].

### 3.6. Drug-Induced Lupus

Certain medications can induce lupus-like symptoms, accompanied by positive ANA results. Distinguishing drug-induced lupus from idiopathic SLE is essential for appropriate intervention. Discontinuation of the implicated medication often leads to the resolution of symptoms and ANA titers [20,21].

### 3.7. Infections and Other Autoimmune Conditions

Infections, such as viral hepatitis and human immunodeficiency virus (HIV), can evoke positive ANA results. Additionally, other autoimmune conditions like autoimmune hepatitis, inflammatory myopathies, and mixed connective tissue disease (MCTD) should be considered in the differential diagnosis [22].

### 3.8. Overlap Syndromes

Some individuals may exhibit features of multiple autoimmune diseases, leading to overlap syndromes. These cases require meticulous evaluation to discern the predominant disease and guide appropriate therapeutic strategies [23].

In navigating the complex landscape of positive ANA patients, the differential diagnosis extends beyond autoimmune diseases to encompass a spectrum of conditions with potential ANA positivity. Clinical correlation, incorporation of specific autoantibody profiles, and adherence to established diagnostic criteria are pivotal for accurate disease classification and subsequent management. The nuanced approach to differential diagnosis ensures that individuals with positive ANA results receive tailored and timely interventions, optimizing outcomes in the diverse array of health conditions associated with ANA positivity.

## 4. ANAs and Cancer: A Complex Interplay

As we navigate the expansive terrain of antinuclear antibodies (ANAs), their significance transcends the boundaries of autoimmune diseases, reaching into the intricate realm of cancer. Initially recognized for their association with autoimmune disorders such as systemic lupus erythematosus (SLE) and scleroderma, ANAs have emerged as enigmatic players in the complex landscape of tumorigenesis. The interplay between ANAs and cancer introduces a multifaceted narrative, challenging traditional perceptions and prompting a deeper exploration of their role as potential markers and influencers in oncological contexts [5].

The detection of ANAs traditionally involved immunofluorescent techniques, notably utilizing human epithelial cell lines (HEp-2) as substrates. These techniques unravel distinct staining patterns that act as fingerprints, indicative not only of autoimmune processes but also of potential connections to malignancies. Efforts to standardize classification and nomenclature of these patterns, such as the international consensus on ANA patterns (ICAP), have been pivotal in enhancing our understanding of their implications in various diseases, including cancer [24].

Studies have progressively unveiled the intriguing association between ANAs and cancer, challenging the conventional belief that these antibodies are exclusive to autoimmune contexts [25]. The presence of ANAs preceding cancer diagnoses has been particularly notable, with certain studies showcasing their occurrence in lung and colon cancer patients before the manifestation of overt clinical symptoms. This pre-existence of ANAs raises intriguing questions about their potential as early markers or indicators of certain malignancies, heralding a paradigm shift in their clinical relevance [26].

However, the relationship between ANAs and cancer is far from straightforward. While some studies suggest a correlation between ANAs and improved survival rates in specific cancer types, their presence also poses challenges. ANAs can lead to autoimmune manifestations, complicating clinical diagnoses and introducing complexities in patient management. This dual nature of ANAs in cancer patients underscores the complexity of their roles in both autoimmune conditions and malignancies [27].

Specific ANA staining patterns have come under scrutiny for their potential associations with particular cancers. For instance, antibodies conventionally linked to SLE, such as anti-dsDNA, have surfaced in various cancers, including bronchogenic carcinoma and lung cancer’s malignant pleural effusions. Notably, these antibodies have displayed potential prognostic significance, particularly in predicting thymoma relapse or serving as biomarkers in colorectal cancer [24].

Moreover, the exploration extends to other intricate staining patterns like anti-DFS70, anti-centromere, anti-Ro/SSA, anti-La/SSB, and anti-Sm antibodies, each elucidating their associations with certain diseases and their potential roles as prognostic markers or therapeutic predictors in different malignancies. The evolving understanding of ANAs in cancer holds promising implications for diagnosis, treatment, and prognosis, underscoring the urgent need for comprehensive research to uncover the precise mechanisms and roles of ANAs in cancer pathogenesis [24,25].

This enigmatic intersection between ANAs and cancer introduces a dynamic layer to our comprehension of both autoimmune and oncological processes. The antibodies, traditionally perceived through the lens of autoimmune diseases, emerge as potential biomarkers and influential factors in the context of cancer. The complexity of their roles, intertwined with the intricacies of tumorigenesis, invites further exploration, and underscores the need for tailored approaches to deciphering the dual impact of ANAs in autoimmune conditions and cancer [27].

In unraveling the complexities of ANAs and their association with cancer, research has identified specific antibodies that sporadically correlate with solid tumors. One such example is the presence of anti-RNP antibodies, which in certain case studies have shown correlation with metastatic undifferentiated carcinoma—a rare childhood malignancy previously unassociated with anti-RNP antibodies [28].

Additionally, studies have highlighted a notable link between anti-RNA polymerase III antibodies and an elevated risk of cancer in patients with systemic sclerosis (SSc). These antibodies have been significantly associated with an increased likelihood of concomitant malignancy, leading researchers to recommend regular cancer screening for individuals testing positive for anti-RNA polymerase III antibodies in the context of SSc [29,30].

Exploring various staining patterns, such as AC-13 (related to cell proliferation), AC-14 (centromere-related), and AC-29 (associated with topoisomerase I DNA), has revealed their implications in cancer scenarios. These patterns have shown potential as markers for specific subgroups of lymphomas and systemic sclerosis with visceral involvement [31].

NuMA (AC-26) proteins have drawn attention due to increased levels observed in patients diagnosed with meningioma, suggesting a unique underlying cause for this particular tumor type. Actin-associated proteins have shown associations with breast cancer, while research has indicated the potential tumor-suppressive role of transgelin in prostate cancer [17].

Moreover, antibodies like anti-ribosomal P protein (seen in SLE) [32], anti-Jo1 (linked to antisynthetase syndrome and polymyositis) [33], and AMA (antimitochondrial antibodies) have shown varied connections to cancer [34]. From being prevalent in lupus patients with active disease to their presence in specific tumors like salivary neoplasms or primary biliary cirrhosis, these antibodies have exhibited diverse associations across different malignancies and autoimmune conditions.

In this evolving landscape of ANAs and cancer, the role of these antibodies extends beyond mere markers. They become dynamic players, potentially influencing the course of malignancies, and offering insights into the underlying mechanisms of tumorigenesis. While the clinical implications are promising, challenges persist in accurately differentiating these antibodies in healthy individuals or those with rheumatic diseases. Further research is essential to fully understand the precise roles of these autoantibodies in cancer diagnosis, prognosis, and response to emerging immunotherapies.

## 5. New Technologies and Challenges in Clinical Interpretation

Specialized studies in the field of antinuclear antibodies (ANAs) have become essential in advancing our comprehension of these complex autoantibodies. The progress in immunofluorescence techniques is notable, as it enables a more detailed characterization of ANA patterns. By utilizing advanced microscopy and staining techniques, these investigations have discovered small differences in patterns, which have helped to create a more detailed classification of ANA-associated autoimmune disorders. The International Consensus on ANA Patterns (ICAP) has been established as a result of these efforts, offering a consistent system of naming that improves the accuracy of reporting and interpretation [7]. In addition to recognizing patterns, these investigations have explored the identification of particular ANA subtypes that are linked to specific autoimmune diseases. For instance, the existence of anti-double-stranded DNA (anti-dsDNA) antibodies strongly suggests the presence of systemic lupus erythematosus (SLE), whereas anti-Ro and anti-La antibodies are frequently associated with Sjögren’s illness. The presence of subtype-specific connections greatly enhances the accuracy of disease diagnoses and facilitates the creation of treatment methods that are specifically tailored to target those subtypes [35].

Longitudinal studies have investigated the temporal dynamics of ANAs, revealing fascinating patterns. Certain individuals may display temporary ANA positive results, underscoring the importance of meticulous interpretation and association with clinical symptoms. Gaining knowledge about the temporal characteristics of the existence of ANA (antinuclear antibodies) enhances the ability to make a more detailed evaluation of autoimmune processes and the likelihood of illness progression [36]. Furthermore, thorough examinations of less prevalent ANA patterns have unveiled their potential therapeutic importance. Common patterns such as homogeneous, speckled, centromere, and nucleolar are thoroughly researched, while less common patterns present diagnostic difficulties. Specialized research focused on these rare patterns seeks to uncover their connections to specific diseases, thus enhancing our overall understanding of the diversity of ANA and its consequences [8].

The examination of ANA prevalence has focused on the differences in geography and population. Investigations of the prevalence of antinuclear antibodies (ANAs) in various communities through epidemiological research enhance our comprehension of the worldwide significance of autoimmune disorders. This research examines genetic, environmental, and lifestyle factors, providing insights into the intricate interaction that influences ANA positive rates in various regions [37]. Moreover, inquiries into the function of ANAs in neurological illnesses have uncovered fascinating associations. ANAs have been detected in disorders such as neuropsychiatric lupus and autoimmune encephalitis, highlighting the possible role of these antibodies in the central nervous system. Comprehending the unique characteristics and consequences of ANAs in neurological situations helps in customizing therapeutic approaches [38].

Turning our attention to the patterns of occurrence and new difficulties in ANA testing, thorough research using data from the National Health and Nutrition Examination Survey (NHANES) in the United States reveals a notable rise in ANA prevalence over a span of 25 years. Between the years 1988 and 1991, as well as 2011 and 2012, the prevalence of the condition increased from 11.0% to 16.1%, indicating a significant rise from about 22.3 million to 41.5 million affected individuals [39,40]. This trend, which is particularly noticeable among teenagers aged 12–19, necessitates additional examination of the reasons that contribute to the higher prevalence of ANA positive results, particularly in younger age groups [41,42]. Nevertheless, ANA testing is not without its difficulties, with the analysis and understanding of outcomes being a key issue. The presence of diverse ANA patterns and titers in both healthy and sick groups adds complexity to the interpretation of results. The Croatian project aims to establish standardized standards for ANA testing, recognizing the significance of focused testing to reduce the likelihood of false-positive results. Nevertheless, differentiating between clinically relevant ANA patterns linked to connective tissue diseases (CTDs) and those observed in healthy individuals or non-rheumatic illnesses remains a challenging undertaking [43]. Technological advancements have brought about new approaches for ANA testing that go beyond the conventional indirect immunofluorescence (IIF) on HEp-2 cells. Although these technologies provide benefits such as automation and increased productivity, they also present obstacles in terms of standardization and consistency of results. It is crucial to ensure consistency among different testing platforms in order to uphold the dependability and medical significance of ANA results [44].

## 6. Future Directions in ANA Research

The trajectory of research on antinuclear antibodies (ANAs) is poised for significant advancements, driven by the ongoing quest to refine diagnostic tools, deepen the understanding of underlying pathogenic mechanisms, and explore novel therapeutic avenues. Future directions are likely to prioritize the development of more sophisticated and specific assays for ANA detection, incorporating cutting-edge technologies to enhance sensitivity and precision. High-throughput techniques, including advanced proteomics and genomics, hold promise in unraveling the complex molecular landscape associated with ANAs. The advent of personalized medicine may play a pivotal role in ANA-related disorders, tailoring treatment strategies based on the specific autoantibody profiles of individual patients. The exploration of the microbiome’s influence on ANA production and autoimmune responses presents an intriguing avenue for research, offering insights into the intricate interplay between host immunity and microbial communities. Artificial intelligence and machine learning applications are anticipated to revolutionize ANA data analysis, facilitating more accurate predictions of disease trajectories, and aiding in early intervention strategies. Collaborative efforts among clinicians, researchers, and technology developers will be crucial in translating these futuristic visions into tangible improvements in ANA-related diagnostics and therapeutic interventions. As the field embraces these innovative approaches, the prospect of more precise ANA detection, tailored treatment regimens, and improved outcomes for individuals with ANA-associated conditions comes into sharper focus.

## 7. Discussion

Insights from multiple authors offer a thorough comprehension of antinuclear antibodies (ANAs), going beyond autoimmune conditions to encompass correlations with cancer. Accurate interpretation of rare ANA patterns is essential for distinguishing between different diagnoses. Specialized research provides vital knowledge about lupus erythematosus and the changing field of ANAs in personalized therapy. The conversation, based on historical backgrounds and the Croatian proposal, looks ahead to future progress, and emphasizes the need for collaborative endeavors in understanding the intricacies of ANAs. Essentially, ANAs have moved beyond their usual role in autoimmune conditions and have become crucial participants in tailored treatment.

## 8. Conclusions

In the realm of antinuclear antibodies (ANAs), our journey through the intricate landscape of diagnostics, clinical significance, and evolving research illuminates a dynamic field at the intersection of immunology, technology, and patient care. ANAs, pivotal in the diagnosis of autoimmune rheumatic diseases, showcase their complexity through diverse staining patterns and associations with a spectrum of health conditions.

The clinical significance of ANAs extends beyond their traditional role in autoimmune diseases, with emerging connections to cancer adding layers to their intricate narrative. The interplay between ANAs and cancer underscores the need for continued exploration, as these antibodies unveil potential roles as early markers and prognostic indicators, shaping the future of both autoimmune and oncological diagnostics.

As we navigate the differential diagnosis in patients with positive ANA results, the importance of a meticulous approach becomes evident. Specific antibody testing, nuanced interpretation of staining patterns, and the recognition of less common ANA patterns contribute to diagnostic precision. The Croatian initiative’s efforts to standardize ANA testing practices exemplify a commitment to enhancing diagnostic accuracy and ensuring consistency across laboratories, fostering a global impact.

Insights from specialized studies shed light on the prevalence and persistence of autoantibodies, particularly in lupus erythematosus, highlighting the importance of a nuanced understanding of these diverse patterns in tailoring patient care.

The future of ANA research beckons with promises of refined diagnostics, personalized medicine approaches, and the integration of cutting-edge technologies. High-throughput omics, the microbiome’s role, and artificial intelligence stand as vanguards in the pursuit of unraveling ANA mysteries, offering a glimpse into a future where precision and personalization define autoimmune diagnostics and treatment.

In conclusion, the multifaceted journey through the world of ANAs underscores the pivotal role these antibodies play in shaping our understanding of health and disease. As research evolves, collaboration and innovation will be the keystones in unlocking the full potential of ANA-related diagnostics, ultimately ushering in an era of enhanced patient care and improved outcomes.

## Data Availability

Not applicable.
